# First case report of spigelian hernia containing the appendix after liver transplantation: Another cause for chronic abdominal pain

**DOI:** 10.1016/j.ijscr.2020.06.003

**Published:** 2020-06-16

**Authors:** Lucas Faraco Sobrado, Lucas Ernani, Daniel Reis Waisberg, Luiz Augusto Carneiro-D’Albuquerque, Wellington Andraus

**Affiliations:** Digestive Organs Transplant Unit, Department of Gastroenterology, Liver Transplantation Division, University of São Paulo School of Medicine, São Paulo, Brazil

**Keywords:** Liver transplantation, Abdominal pain, Amyand hernia, Spigelian hernia, Appendix, Surgery

## Abstract

**Introduction:**

Abdominal ventral hernias are common in chronic liver disease due to increased abdominal pressure and sarcopenia. Following liver transplantation, diagnosis of chronic abdominal pain is challenging because it may relate to immunosuppression, scaring or opportunistic infections.

**Presentation of case:**

A 62 years-old male presented with chronic abdominal pain one year following liver transplantation due to hepatocellular carcinoma. After work-up he was diagnosed with a Spigelian hernia containing the appendix. We did hernia repair with mesh but appendectomy was not performed since it showed no signs of inflammation. On follow-up the patient had complete resolution of the pain.

**Discussion:**

This is the first case of spigelian hernia containing the appendix following liver transplantation. Mesh repair can be safely performed in this setting but incidental appendectomy is controversial due to higher morbidity and mortality. In this case report we discuss the relationship between liver transplantation, abdominal hernias and the pitfalls of incidental appendectomy.

**Conclusion:**

Uncommon ventral hernias are a possible cause for chronic abdominal pain after surgery and should be investigated with imaging studies. Mesh repair is safe but incidental appendectomy in the immunosuppressed is not encouraged due to increased morbidity.

## Highlights

• Hernia repair is associated with higher morbidity in chronic liver disease.
• Chronic abdominal pain following transplantation is a diagnostic challenge.
• Incidental appendectomy in the immunosuppressed carries additional risk.
• Mesh repair can be safely performed following liver transplantation.
• Uncommon ventral hernias can be the source of chronic abdominal pain following liver transplantation.

## 1 Introduction

Abdominal ventral hernias are common before and after liver transplantation. Sarcopenia and increased abdominal pressure in cirrhosis play a role in their development and may persist following transplantation [1]. Up to 43% of the recipients develop incisional hernias [2] and emergence surgery is associated with higher morbidity and mortality [3,4].

Early diagnosis is challenging since symptoms can be subtle and chronic abdominal pain may relate to adhesions, immunosuppression or opportunistic infections. Transplantation may induce metabolic syndrome and weight gain, making physical examination more challenging. Also, due to large incision and immunosuppression, patients are less responsive to painful stimulus. Therefore, diagnosis of abdominal pain post liver transplant is not straightforward and it is important to be aware of differential diagnoses and uncommon clinical scenarios.

This case report has been reported in line the SCARE criteria [5] and it aims to discuss the relationship between liver transplantation, abdominal hernias and the pitfalls of incidental appendectomy.

## 2 Presentation of case

A 62 years-old male with long-term diabetes, hypertension, dyslipidemia and non-alcoholic fatty liver disease (NASH) was diagnosed with hepatocellular carcinoma. His past surgical history included a previous right inguinal hernioplasty with mesh. After complete work-up he was deemed eligible to liver transplantation and surgery was uneventful.

**Fig. 1:**
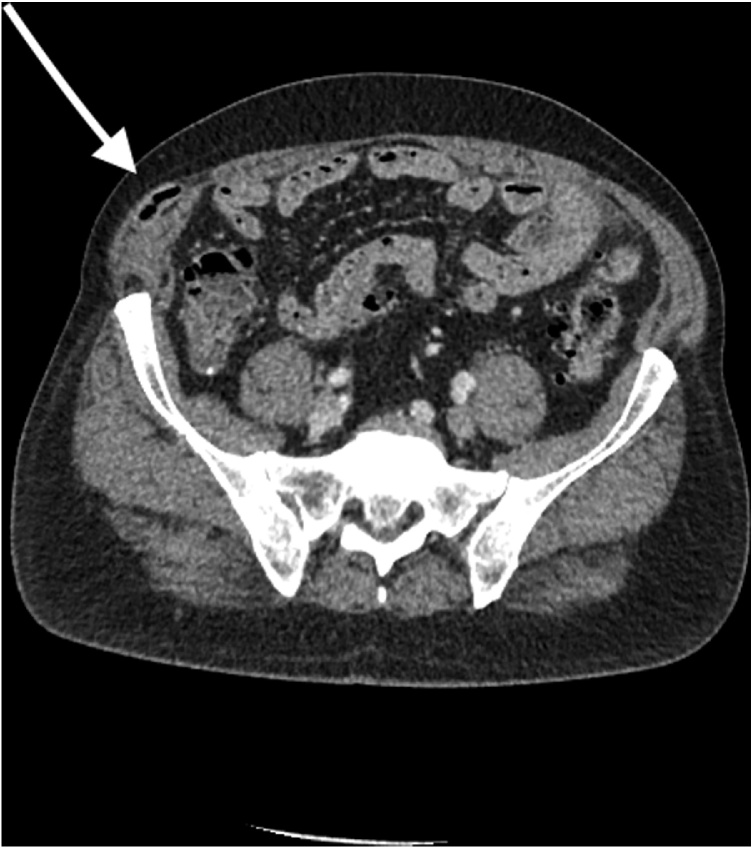
CT Scan showing Spigelian hernia containing the appendix (arrow).

On follow-up the patient was placed on immunosuppression with tacrolimus and remained the use of aspirin, metformin, atorvastatin and gliclazide for control of his clinical comorbidities. During office visits in the first year following surgery, he complained of chronic right-sided abdominal pain exacerbated by movement. Clinical examination was unremarkable and no hernias were detected on previous incisions. We performed a complete medical work-up and abdominal computed tomography that revealed a spigelian hernia containing the appendix (Fig. 1). This finding was not present before liver transplantation.

**Fig. 2:**
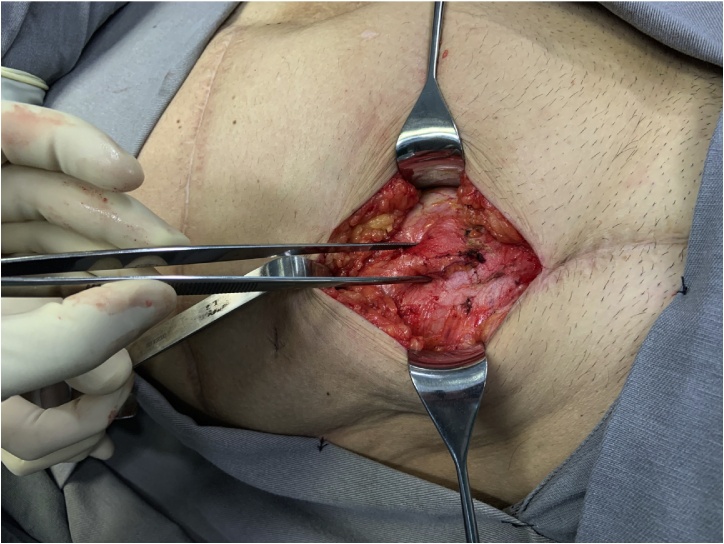
Intact external oblique muscle fascia and intraparietal bulge.

**Fig. 3:**
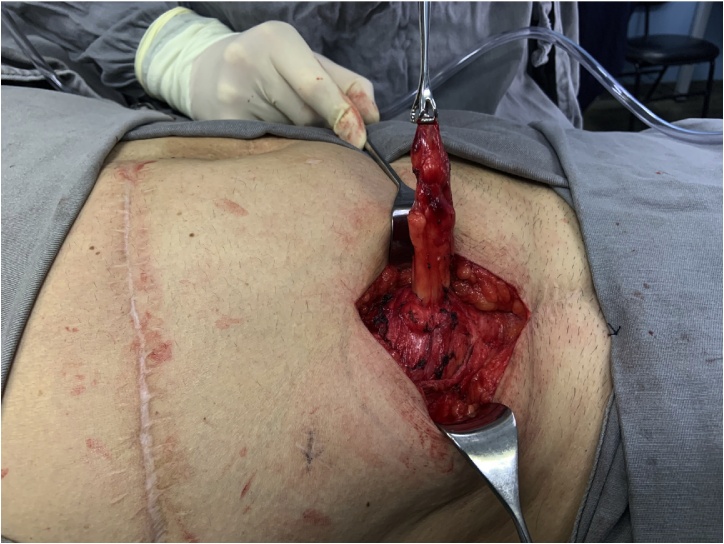
Spigelian hernia containing the appendix.

**Fig. 4:**
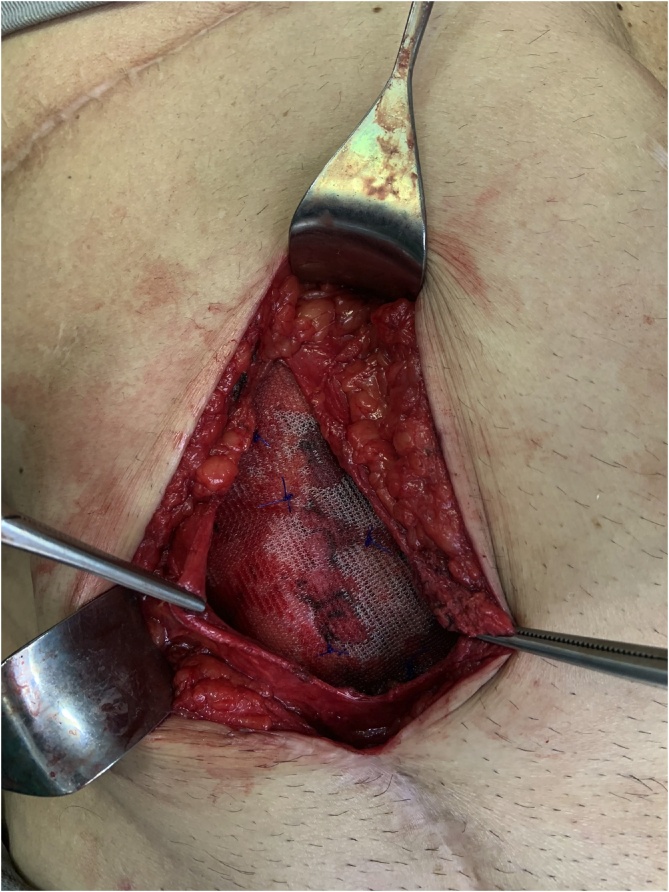
Hernia repair with polypropylene mesh below the external oblique fascia.

The patient was listed for elective surgery, which was performed by the authors LFS and LE. At the operation, an oblique incision was made and after opening the skin and subcutaneous, a bulge just below the fascia of the external oblique was noticed (Fig. 2). Opening of the fascia revealed an intraparietal appendix, consistent with spigelian hernia (Fig. 3). The hernia sac was 2 × 2 cm. Hernia repair was performed with an 8 × 8 cm polypropylene mesh just below the fascia of the external oblique muscle (Fig. 4). The appendix had no signs of inflammation so appendectomy was not performed. Post-operative course was uneventful and he was discharged on day two. On follow-up, he was very satisfied with the procedure, had complete resolution of abdominal pain and no signs of recurrence.

## 3 Discussion

To our knowledge this is the first case of spigelian hernia (SH) containing the appendix after liver transplantation, which could result in higher morbidity and mortality if not promptly diagnosed. Previous reports included mostly patients with acute complications, such as incarceration and acute appendicitis [6,7]. In one case, mesh repair was not possible due to gross contamination and necrosis [8].

SH was described in 1764 by Josef Klingosh and named after the anatomist Adrian Spieghel. The true prevalence is uncertain but may be up to 2% of abdominal hernias. The apparent incidence has increased over the last decades, possibly due to the increased incidence of obesity and the accuracy of diagnostic imaging [9]. SH diagnosis is challenging because the fascia of the external oblique muscle is intact, so physical examination is often unremarkable. The small diameter of the fascia defect makes them prone to incarceration and in some series up to 25% patients were treated with emergency surgery [10].

The content of the hernia sac is variable: omentum, small bowel, colon, epiploic appendage have all been described. SH containing the appendix is a rare entity and only few cases have been reported in the literature. This hernia type lacks an eponym and is considered a different entity from Amyand hernia, which corresponds to an inguinal defect with the appendix.

Incisional hernias after liver transplantation are not uncommon and have been reported in up to 43% of patients [2]. Fikatas et al. after 810 liver transplants found an incidence of 15% and showed that higher BMI, older age and diabetes mellitus were all independent risk factors [11]. A recent review of 19 studies demonstrated other important risk factors, such as: immunosuppression, reoperation, transverse incision with midline extension [12]. In our case, however, the patient did not have incisional hernia or acute complication and was diagnosed due to chronic abdominal pain. Surgery was performed in an elective setting and intraoperative findings revealed a normal appendix so appendectomy was not performed.

Incidental appendectomy remains controversial but it is reasonable to operate on young and fit patients with low morbidity. However, it is not recommended to operate on the elderly and the immunosuppressed due to increased risk of complications and the lower chances of developing acute appendicitis in older age. Kotaluoto et al. analyzed appendectomies performed for over two decades in Finland and observed a 39-fold increase in mortality among the elderly [13].

It is important to notice that acute appendicitis in immunosuppressed patients will often not present with the most common symptoms and the clinical picture may be misleading. Fortunately, our patient was diagnosed before any complication ensued.

## 4 Conclusion

Chronic abdominal pain following liver transplantation is a challenging diagnosis. Uncommon ventral hernias are a possible cause for chronic abdominal pain after surgery and should be investigated with imaging studies since physical examination alone is unreliable. In the context of immunosuppression, mesh repair is safe but incidental appendectomy is not encouraged due to increased morbidity and mortality.

## Declaration of Competing Interest

There is no relevant conflict of interest.

## Sources of funding

We did not receive any funding for this case report.

## Ethical approval

Ethical approval exemption was given for this study.

## Consent

Written informed consent was obtained from the patient.

## Author contribution

Lucas Faraco Sobrado: writing - original draft, review and editing.

Lucas Ernani: writing - original draft, review and editing.

Daniel Reis Waisberg: writing - original draft, review and editing.

Luiz Augusto Carneiro D’Albuquerque: writing - review and editing, supervision.

Wellington Andraus: writing - review and editing, supervision.

## Registration of research studies

Not applicable.

## Guarantor

Lucas Faraco Sobrado.

## Provenance and peer review

Not commissioned, externally peer-reviewed.
